# The EAG Voltage-Dependent K^+^ Channel Subfamily: Similarities and Differences in Structural Organization and Gating

**DOI:** 10.3389/fphar.2020.00411

**Published:** 2020-04-15

**Authors:** Francisco Barros, Pilar de la Peña, Pedro Domínguez, Luisa Maria Sierra, Luis A. Pardo

**Affiliations:** ^1^ Departamento de Bioquímica y Biología Molecular, Universidad de Oviedo, Edificio Santiago Gascón, Oviedo, Spain; ^2^ Departamento de Biología Funcional (Area de Genética), Instituto Universitario de Oncología del Principado de Asturias (IUOPA), Instituto de Investigación Sanitaria del Principado de Asturias (ISPA), Universidad de Oviedo, Oviedo, Spain; ^3^ Oncophysiology Group, Max Planck Institute of Experimental Medicine, Göttingen, Germany

**Keywords:** potassium channel, EAG family, voltage-dependent gating, cytoplasmic domains, allosteric gating, structure-function relationships

## Abstract

EAG (*ether-à-go-go* or *KCNH*) are a subfamily of the voltage-gated potassium (Kv) channels. Like for all potassium channels, opening of EAG channels drives the membrane potential toward its equilibrium value for potassium, thus setting the resting potential and repolarizing action potentials. As voltage-dependent channels, they switch between open and closed conformations (gating) when changes in membrane potential are sensed by a voltage sensing domain (VSD) which is functionally coupled to a pore domain (PD) containing the permeation pathway, the potassium selectivity filter, and the channel gate. All Kv channels are tetrameric, with four VSDs formed by the S1–S4 transmembrane segments of each subunit, surrounding a central PD with the four S5–S6 sections arranged in a square-shaped structure. Structural information, mutagenesis, and functional experiments, indicated that in “classical/*Shaker*-type” Kv channels voltage-triggered VSD reorganizations are transmitted to PD gating *via* the α-helical S4–S5 sequence that links both modules. Importantly, these *Shaker*-type channels share a domain-swapped VSD/PD organization, with each VSD contacting the PD of the adjacent subunit. In this case, the S4–S5 linker, acting as a rigid mechanical lever (electromechanical lever coupling), would lead to channel gate opening at the cytoplasmic S6 helices bundle. However, new functional data with EAG channels split between the VSD and PD modules indicate that, in some Kv channels, alternative VSD/PD coupling mechanisms do exist. Noticeably, recent elucidation of the architecture of some EAG channels, and other relatives, showed that their VSDs are non-domain swapped. Despite similarities in primary sequence and predicted structural organization for all EAG channels, they show marked kinetic differences whose molecular basis is not completely understood. Thus, while a common general architecture may establish the gating system used by the EAG channels and the physicochemical coupling of voltage sensing to gating, subtle changes in that common structure, and/or allosteric influences of protein domains relatively distant from the central gating machinery, can crucially influence the gating process. We consider here the latest advances on these issues provided by the elucidation of eag1 and erg1 three-dimensional structures, and by both classical and more recent functional studies with different members of the EAG subfamily.

## Overview

**Figure 1:**
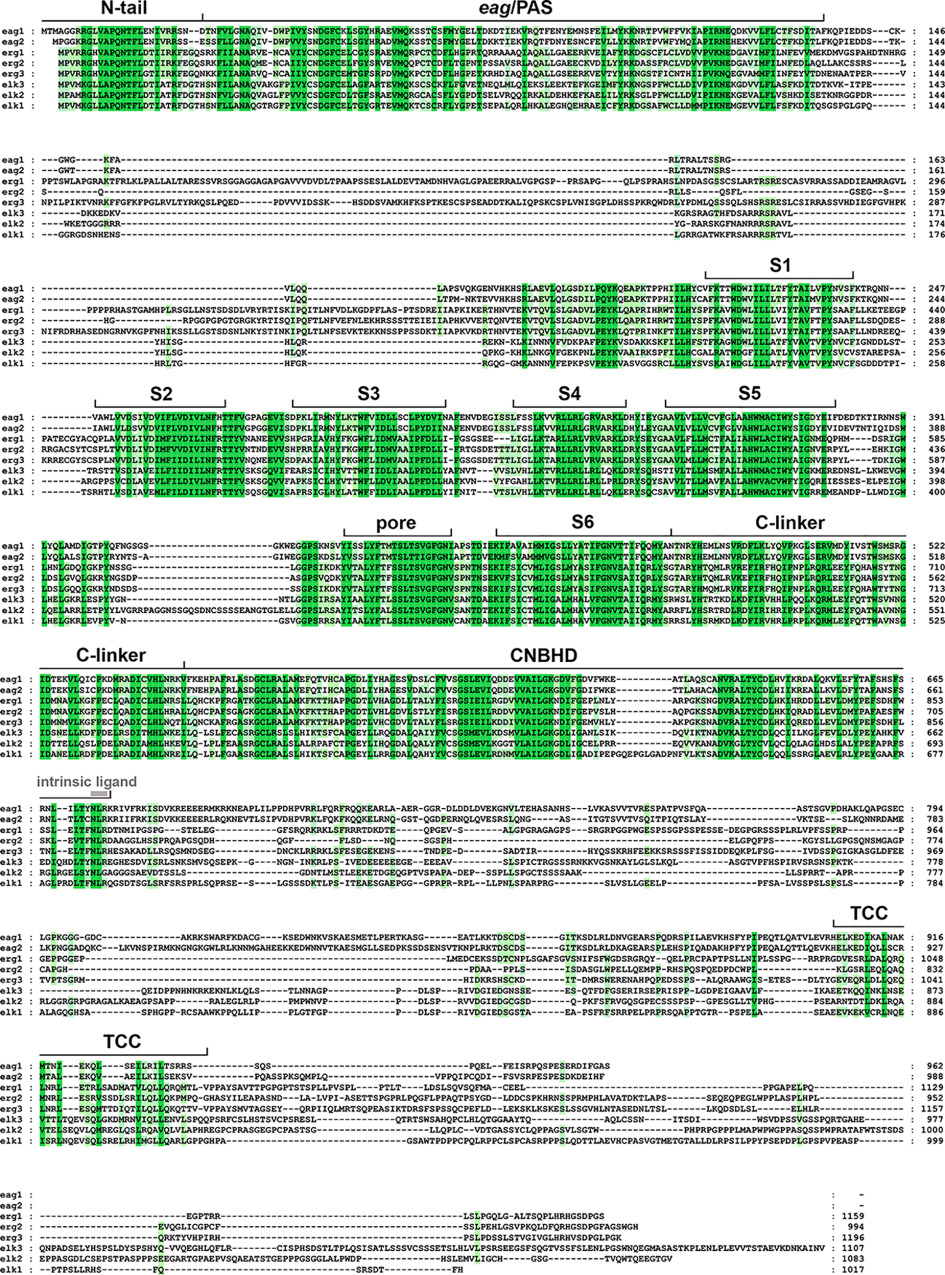
Amino acid sequence alignment of the human *ether-á-go-go* (EAG) channels polypeptides. The alignment was generated using Clustal Omega (https://www.ebi.ac.uk/Tools/msa/clustalo/) and analyzed and edited using GeneDoc software. Gaps required to optimize the alignment are shown as *dashes*. Identical or highly similar residues in *all* the EAG sequences are shadowed *dark green*, and residues conserved in *most* sequences are shadowed in *light green*. The boundaries of the transmembrane helices S1–S6, pore helix (pore), and intracellular domains [*P*er-*A*rnt-*S*im (PAS) domain preceded by the initial N-tail, C-linker, cyclic nucleotide-binding homology domain (CNBHD), and a carboxy terminal proposed tetramerization coiled-coil/TCC] are indicated above the sequence. The position of the residues corresponding to the intrinsic ligand of the CNBHD is also indicated using grey letters. The accession numbers for the polypeptide sequences are: eag1, NP_002229.1; eag2, NP_647479.2; erg1, NP_000229.1; erg2, 110406.1; erg3, NP_150375.2; elk3, NP_653234.2; elk2, NP_036416.1; elk1, NP_036417.1.

**Figure 2:**
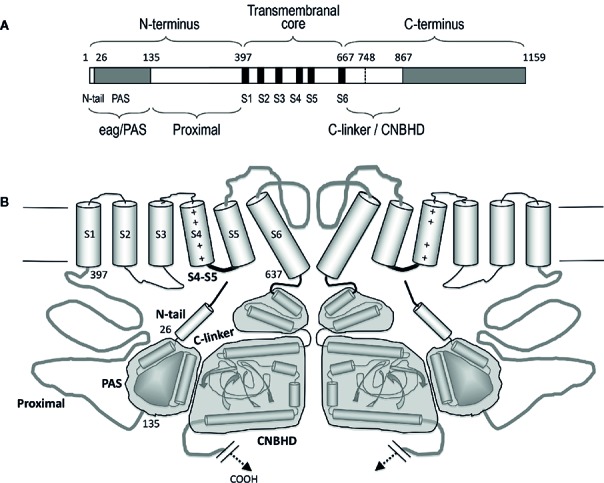
Schematic representation of the structural organization of the erg1 (Kv11.1/hERG) channel. **(A)** Schematic linear diagram of an erg1 channel subunit. The six transmembrane helices (S1 to S6) are represented as black boxes. The position of the boundaries between the different channel domains denoted above and below the bar is indicated with numbers. The size of each region is represented approximately proportional to scale. **(B)** Schematic cartoon representing two α-subunits of a four-subunit erg1 channel tetramer. The S1–S4 transmembrane helical segments that make up the voltage sensor domain are linked *via* an S4–S5 linker (thick black line) to the pore-forming domains (segments S5 and S6 and intervening pore loop, thick grey line). Note the short length of the S4–S5 linker associated to the non-domain-swapped organization of the transmembranal core. Both the N- and the C-terminal regions are intracellular. At the N-terminus, the amphipatic helix (residues 10–23) that follows the initial flexible segment in the N-tail, and the PAS homologous domain (residues 26–134), are depicted as a small cylinder and a globular grey structure, respectively. The erg1 exclusive proximal domain of the N-terminus (residues 135–397) is represented as a grey line connected to the S1 helix. At the C-terminus, the C-linker and cyclic nucleotide-binding homology domain (CNBHD) domains are schematized, encompassed in grey blocks, as a pair of cylinders and as cylinders and sheets, respectively. The long distal region of the carboxy terminus that remains structurally uncharacterized and with no recognized direct influence in channel gating, has been omitted for clarity.

*KCNH* (EAG or *ether-á-go-go*) channels constitute a subfamily of the voltage-gated family of potassium (Kv) channels (Gutman et al., 2005). Three subtypes (Kv10 to Kv12) are included in this group: (i) eag (Kv10) with two mammalian members, Kv10.1 or eag1 and Kv10.2 or eag2, encoded by the *KCNH1* and *KCNH5* genes, respectively; (ii) erg (eag-related gene or Kv11) comprising three channels, Kv11.1 or erg1, Kv11.2 or erg2, and Kv11.3 or erg3, encoded by *KCNH2*, *KCHN6*, and *KCNH7*; and (iii) elk (eag-like K^+^ channel or Kv12) including Kv12.1 or elk1, Kv12.2 or elk2, and Kv12.3 or elk3, corresponding to genes *KCNH8*, *KCNH3*, and *KCNH4*. All of them present substantial sequence homology with the other Kv channel subfamilies (Kv1 to Kv9), and share a common primary structure organization (**Figure 1**). In addtion, they show a tetrameric assembly in which each subunit contributes its six transmembrane segments (S1-S6) to form a transmembrane core. **Figure 2** depicts a schematic representation of the general topology of these channels, exemplified by that of erg1 (Kv11.1/hERG). The schemes try to provide a better visual guidance through the structural and functional regions recognized in the channel, but do not recapitulate the whole set of interactions between them or their possible dynamic reorganizations during the gating process, as discussed below. Segments S1 to S4 form the voltage-sensing domain (VSD) of each subunit; S4 contains several positively charged residues and provides the main transmembrane voltage-sensing component. The two additional transmembrane helices (S5 and S6), plus the intervening pore loops of the four subunits, associate to form the tetrameric structure that surrounds a central conduction pathway, and constitutes the ion permeation pore-gate domain (PD) (Yellen, 1998; Yellen, 2002; Swartz, 2004; Yu et al., 2005; Ashcroft, 2006; Swartz, 2008; Bezanilla, 2008). To this common protein core different cytoplasmic modules (and accessory subunits), able to strongly influence the gating and other functional properties, have been added through evolution, (reviewed in Barros et al., 2012). A characteristic feature of EAG channels is the presence of long cytoplasmic N- and C-terminal ends, that include some structures not found in any other Kv channel type. Thus, in the amino terminus they contain an initial section conserved among all members of the group (**Figure 1**), named for this reason “eag domain” and that, due to the presence of a PAS (*P*er-*A*rnt-*S*im) region typical of the large family of the “PAS domain proteins”, is also frequently called the “eag/PAS domain”. Importantly, upstream this PAS region, there is a stretch of around 25 amino acids that appeared disordered and therefore unresolved in the initial high-resolution crystal structures of the erg1, eag1, and *Drosophila* elk eag/PAS domains (Morais-Cabral et al., 1998; Adaixo et al., 2013). This initial section of the protein has been named N-terminal tail or N-tail (Ng et al., 2011; De la Peña et al., 2011; Vandenberg et al., 2012; De la Peña et al., 2013; Barros et al., 2018), N-CAP (Gustina and Trudeau, 2012; Morais-Cabral and Robertson, 2015) or PAS-loop (Whicher and MacKinnon, 2019), and is composed of an initial short flexible segment, followed by an amphipatic alpha-helix (Gustina and Trudeau, 2012; Barros et al., 2012; Vandenberg et al., 2012; Morais-Cabral and Robertson, 2015; Whicher and MacKinnon, 2016; Wang and MacKinnon, 2017). Remarkably, the flexible N-tail could play specific role(s) on channel gating, independent from those of the PAS domain itself (Barros et al., 2012; Vandenberg et al., 2012; Gustina and Trudeau, 2012; Morais-Cabral and Robertson, 2015. See below). Interestingly, some of the cytoplasmic EAG channels sections display a high degree of homology with other domains from channels outside the Kv family [e.g., the cyclic nucleotide-gated (CNG), the hyperpolarization-activated and cyclic nucleotide-gated (HCN) and some inwardly rectifying plant K^+^ channels], all of them pertaining to the named “S4” or “6TM1P” group of the pore-loop channel family, but having different selectivity, or no or even inverted voltage dependence (Gutman et al., 2005; Yu et al., 2005; Ashcroft, 2006; Lau et al., 2018). Indeed, the presence of intracellular domains either able to bind cyclic nucleotides (cyclic nucleotide-binding domain, CNBD), or sharing high structural homologies with those domains but unable to bind nucleotides (cyclic nucleotide-binding homology domain, CNBHD), has allowed classifying the EAG, CNG, and HCN channels under the named CNBD channel family (James and Zagotta, 2018), even though the voltage-dependence, selectivity, and cyclic nucleotide regulation of the EAG channels are different from those of the CNG and HCN channels (James and Zagotta, 2018; Barros et al., 2019).

We will consider here the differential properties exhibited by some of the EAG channels at the functional level, in order to establish possible structure-function correlations centered in their perhaps more conspicuous property, the voltage-dependent gating. Furthermore, we will summarize some of the most recent contributions to our knowledge of the molecular basis of EAG channel gating, mainly fueled by functional data from channel variants (S4–S5 split channels) lacking a covalent link between the VSD and the PD at the level of the S4–S5 linker, as well as by the recent cryo-electron microscopy (cryo-EM) elucidation of the three-dimensional structure of some EAG channels. Finally, we will consider some possible limitations of these studies and future directions to further advance this topic.

## EAG Channels: Prototypic Examples of Non-Domain-Swapped Channel Core Architecture

**Figure 3:**
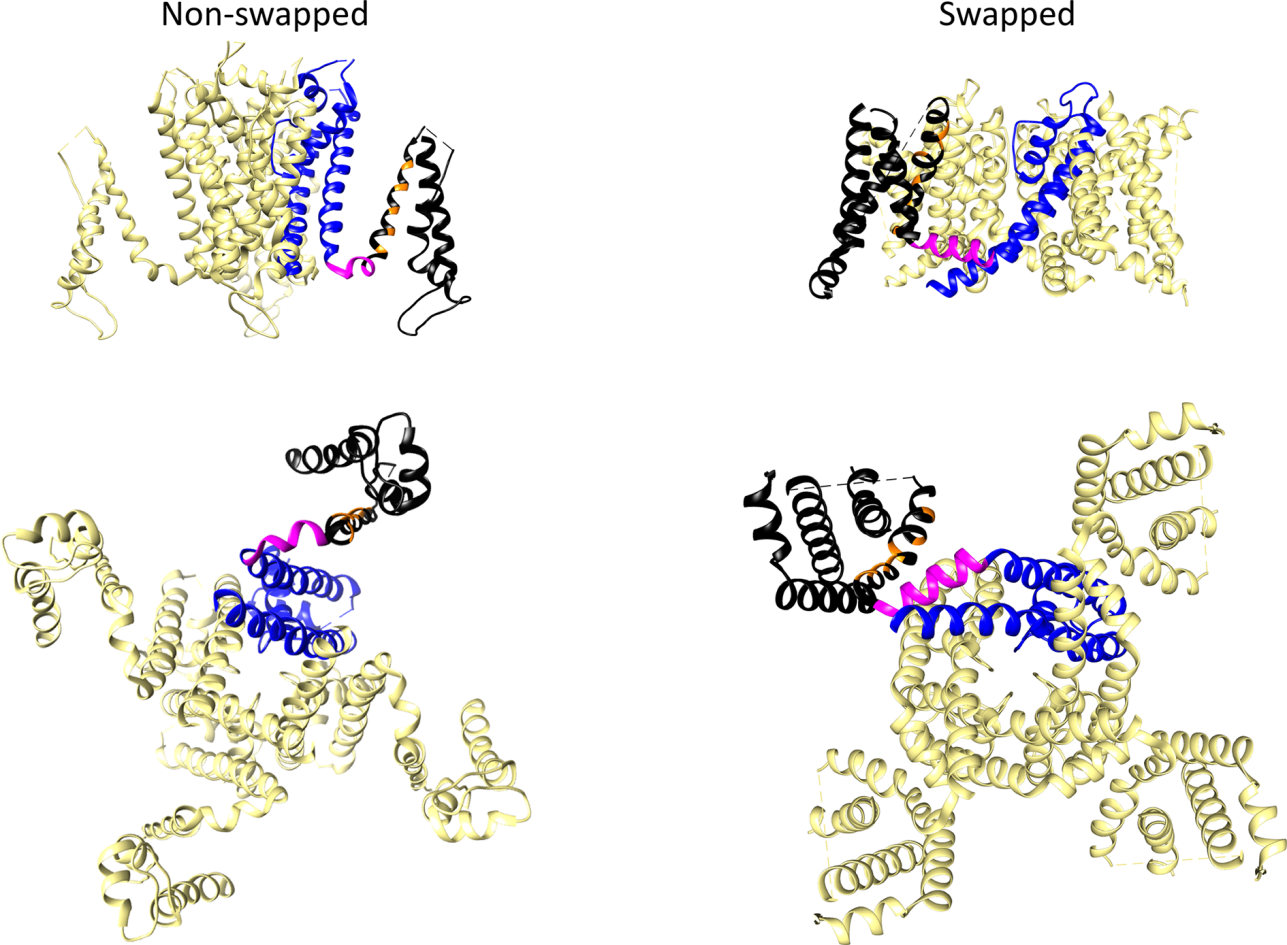
Comparison of non-domain-swapped and domain-swapped architectures of Kv channels. Left, erg1 (Kv11.1/hERG, PDB: 5VA2) non-domain swapped structure. Right, Kv1.2 (PDB: 2A79) domain-swapped structure. Only the transmembrane core domains are depicted viewed from the membrane plane (top) and from the cytoplasmic side (bottom). One of the subunits is shown coloured with the pore domain (PD) structures in blue, the S4–S5 linker in magenta, and the voltage sensing domain (VSD) in black. Orange is used to mark the position of positively charged residues in the S4 transmembrane helix of the VSD.

The three-dimensional protein structures of many ion channels, including some EAG subfamily channels and other members of the structurally-related CNBD family have been elucidated, initially using X-ray crystallography and NMR spectroscopy, and currently, by the spectacular improvements in single particle cryo-EM (reviewed in Vandenberg et al., 2017; Lau et al., 2018; James and Zagotta, 2018; Okamura and Okochi, 2019; Barros et al., 2019). The discovery that, despite their shared common primary organization, the EAG channels and other members of the Kv family can adopt two main architectural patterns in their transmembrane core (**Figure 3**), caused an essential breakthrough in our view of the structural basis of the molecular mechanism(s) involved in the voltage-triggered gating of these entities.

In many Kv channels, the peripheral VSDs contact the PD of a neighboring subunit, leading to a domain-swapped architecture (**Figure 3**). In this case, a long α-helical linker (called the S4–S5 linker) covalently connects the last residue of the S4 helix of the VSD with the first one in the S5 helix of the PD, and cuffs around the helical bundle formed by the S6 helices that usually constitutes the cytoplasmic channel gate (Holmgren et al., 1998; Del Camino et al., 2000; Mitcheson et al., 2000; Del Camino and Yellen, 2001; Witchel, 2004; Webster et al., 2004; Del Camino et al., 2005; Swartz, 2005; Boulet et al., 2007; Wynia-Smith et al., 2008; Thouta et al., 2014). This is typical of the Kv1 to Kv9 (*Shaker*-type) subfamilies, but also occurs in the Nav and Cav voltage-gated sodium and calcium channels (Wisedchaisri et al., 2019), and in the channels from the transient receptor (TRP) family, which are structurally related to Kv channels but are generally non-selective among cations and in most cases voltage-independent (reviewed in Barros et al., 2019). There are indications that in the domain-swapped and voltage-dependent type of channels, an electromechanical coupling between the VSD and the gate exists, in which the S4–S5 linker, acting as a mechanical lever, transmits to the cytoplasmic gate the force generated by the VSD conformational rearrangements, leading to channel activation and/or deactivation (Long et al., 2005; Labro et al., 2008; Vardanyan and Pongs, 2012; Blunck and Batulan, 2012; Jensen et al., 2012; Chowdury et al., 2014; Kalstrup and Blunck, 2018).

In contrast to this architecture, in channels that display a very short and non α-helical S4–S5 linker, the VSD contacts the PD of the same subunit, inducing a non-domain-swapped organization of the transmembrane core (**Figure 3**). This structural characteristic was first demonstrated in the Kv10.1 (eag1) channel (Whicher and MacKinnon, 2016) and subsequently confirmed for Kv11.1 (erg1), Slo1 and SK Ca^2+^-activated K^+^ channels, the Na^+^-dependent K^+^ channel Slo2.2, the HCN1 hyperpolarization-activated and cyclic nucleotide-gated cation channel, and the prokaryotic and eukaryotic cyclic-nucleotide-gated (CNG) channels (Hite et al., 2015; Wang and MacKinnon, 2017; Hite et al., 2017; Tao et al., 2017; Lee and MacKinnon, 2017; James et al., 2017; Li et al., 2017; Lee and MacKinnon, 2018). Note, however, that although a short S4–S5 linker may determine a non-swapped organization, such architecture is also present in the SK channels even though they contain a quite long S4–S5 with two α-helices (Lee and MacKinnon, 2018), and that a domain-swapped organization has been observed in TRPM8 channels showing no obvious or only a short S4–S5 linker (Yin et al., 2018). Strikingly, recent cryo-EM data indicate that in this case the transition between the closed conformation (lacking a canonical S4–S5 linker) and a desensitized conformation through binding of calcium induces a rearrangement of the S4–S5 into a typical α-helical S4–S5 linker architecture. However, in both cases a similar domain-swapped organization of the transmembrane channel core is maintained (Diver et al., 2019). Also, it has been reported that introducing a single point mutation in the S5 transmembrane helix of the TRPV6 channel converts the domain swapped organization to a non-domain swapped yet still functional one (Singh et al., 2017). Noticeably, the domain-swapped variant can be also converted to a non-domain-swapped conformation by shortening the S4–S5 linker, although in this case a non-functional channel is generated. This fact indicates that whereas a short length of the S4–S5 linker is indeed a critical determinant of a non-swapped architecture, other structural variations can also trigger alterations of swapping, associated or not to perturbations in gating function. This finding has been interpreted as an indication that, apart from differences in S4–S5 length, transmembrane domain arrangement can be altered under a variety of conditions, including functional and/or dynamic reorganizations of channel structure (Singh et al., 2017). This interpretation also opens the possibility that other channel regions relatively distant from the central gating machinery, may contribute to the structural organization of the protein. Note also that in many of the non-domain swapped channels gating is controlled by the binding of diverse ligands to intracellular regions that show an extensive domain swapping between the tetramer subunits (Stevens et al., 2009; Gustina and Trudeau, 2011; Gianulis et al., 2013; Haitin et al., 2013; Ng et al., 2014; Whicher and MacKinnon, 2016; Wang and MacKinnon, 2017; Zhao et al., 2017; Barros et al., 2019). In any case, it is clear that in non-domain-swapped, but genuinely voltage-dependent Kv channels, such as Kv10.1 and Kv11.1, the short length and organization of the S4–S5 linker would not allow it to act as a rigid mechanical lever able to pull apart the N-terminal portion of S5 and the C-terminal end of S6 to open the cytoplasmic channel gate, as it seems to happen in the canonical voltage-gated K^+^ channels (Long et al., 2005; Labro et al., 2008; Vardanyan and Pongs, 2012; Blunck and Batulan, 2012; Jensen et al., 2012; Chowdury et al., 2014; Kalstrup and Blunck, 2018). Indeed, pioneer studies with Kv10.1 and Kv11.1 channels in which the separate N- and C-terminal halves of the protein were expressed after breaking the covalent continuity of the S4–S5 linker (S4–S5 split channels), demonstrated the production of voltage-gated channels exhibiting voltage-sensing and permeation properties similar to those of the complete protein (Lorinczi et al., 2015). Thus, both structural and functional evidences point to the existence of a new mechanism in this type of Kv channels, different to the classical lever one proposed for the *Shaker*-like Kv channels (Whicher and MacKinnon, 2016; Wang and MacKinnon, 2017; Tao et al., 2017; Flynn and Zagotta, 2018; Zhao et al., 2017; Lee and MacKinnon, 2018; James and Zagotta, 2018).

It is interesting to note that although the EAG subfamily channels and other members of the so-called CNBD channel group share some common structural features, such as the presence of a VSD and PD arrangement and a non-swapped architecture, this group includes entities exhibiting quite divergent functional and/or gating properties (reviewed in James and Zagotta, 2018; Barros et al., 2019). These divergences are even more evident when other non-domain-swapped channels are considered. Thus, this structural architecture is encountered in channels showing:

- a pure ligand-mediated mechanism of gating with very little or no voltage dependence. In the case of the SK channels, Ca^2+^-bound to calmodulin (Ca^2+^-CaM), associated to a cytoplasmic CaM binding domain (CaMBD), opens the permeation pathway. In the case of Slo2.2, opening is the result of a Na^+^-mediated allosteric regulation of a cytoplasmic gating ring. In the CNG channels, gating depends on the binding of cyclic nucleotides to intracellular CNBDs, that expand the S6 helices bundle and open the cytoplasmic channel gate (Hite et al., 2015; Hite and MacKinnon, 2017; James et al., 2017; Li et al., 2017; Lee and MacKinnon, 2018).
- a dual ligand-voltage regulation of gating, as in BK/Slo1 channels, whose opening is synergistically regulated by both membrane depolarization and intracellular Ca^2+^ increase acting on a cytoplasmic gating ring (Horrigan and Aldrich, 2002; Yang et al., 2015; Jia et al., 2018).
- a voltage-dependent activation with so-called “inverted” gating polarity, as in the HCN channels, in which, unlike the typical depolarization-dependent activation exhibited by the Kv channels, membrane depolarization causes channel closing while activation is triggered by membrane hyperpolarization. In this case, an increased activity is also elicited upon binding of cyclic nucleotides to their intracellular CNBDs (Lee and MacKinnon, 2017; James and Zagotta, 2018).
- a pure voltage- and depolarization-dependent activation gating, as in the genuine Kv channels of the EAG subfamily. Noticeably, even within this channel group, in which strong similarities in primary structure (**Figure 1**) and three-dimensional architecture (Whicher and MacKinnon, 2016; Wang and MacKinnon, 2017) exist, clear differences in gating behavior can also be encountered (Schwarz and Bauer, 2004; Bauer and Schwarz, 2018). These include (i) different ranges of activation and deactivation voltage dependence and diverse gating kinetics, sometimes determined by the potential level previous to the stimulus, (ii) quite different inactivation behavior, and (iii) in the case of the erg (Kv11) channel subtype, gating kinetics inverse to those of other Kv channels, that functionally make them behave as inward-rectifiers (Vandenberg et al., 2012; Bauer and Schwarz, 2018; see next). Altogether, these data suggest that subtle differences in molecular architecture and/or divergences in modulation of the primary gating machinery (allosteric influences), by relatively distant protein domains (e.g., by some cytoplasmic regions showing structural divergence in different members of the same family), can crucially determine the functional output.

## Functional Heterogeneity and Expression Patterns Inside the EAG Channel Subfamily

Expression of different members of the EAG channel subfamily has been observed in a variety of tissues (Ganetzky et al., 1999; Morais-Cabral and Robertson, 2015; Bauer and Schwarz, 2018). All of them are highly expressed in the nervous system, in which they may help to regulate excitability of neuronal cells, although lack of selective blockers of eag and elk channel subtypes mostly precluded proper isolation of native currents in this type of cells, and limited the knowledge of their specific impact on cell excitability (Bauer and Schwarz, 2018). Noticeably, eag1 limits activity-dependent Ca^2+^ entry in presynaptic terminals in the cerebellum, although its function seems to be exclusively exerted in situations with high levels of activity (Mortensen et al., 2015). Nevertheless, a wealth of information has been obtained about the erg1 (hERG) and eag1 physiological roles, due to their recognized expression in heart and tumor cells. Thus, expression of erg1 in cardiac cells is the basis of the fast delayed-rectifier current (I_Kr_), that contributes to termination of cardiac action potentials and determines the appearance of type 2 long-QT (LQT2) syndrome and higher probability of sudden cardiac death, when inherited mutations or pharmacological block cause loss of channel function (Sanguinetti and Tristani-Firouzi, 2006; Vandenberg et al., 2012). In this case, the particular kinetic properties of the erg-mediated currents, and the availability of some pharmacological inhibitors, also allowed to recognize a role of these channels setting the electrical activity in several non-neuronal and non-cardiac cells, such as gastrointestinal smooth muscle fibers, glomus cells of the carotid body, adenohypophysial lactotrophs, epinephrine-releasing chromaffin cells, and pancreatic islet insulin-releasing β-cells and glucagon-releasing α cells (Schwarz and Bauer, 2004; Wanke and Restano-Cassulini, 2007; Vandenberg et al., 2012; Barros et al., 2012; Babcock and Li, 2013; Bauer and Schwarz, 2018). Additionally, both erg1 and eag1 channel expression is aberrantly increased in a variety of tumor cells, where they increased cell proliferation level and tumor malignancy (Arcangeli et al., 2009; Pardo and Stühmer, 2014; Lastraioli et al., 2015; Prevarskaya et al., 2018).

Apart from their presence and putative role in different native tissues, all EAG channel subtypes have been heterologously expressed and their functional properties subsequently characterized. Thus, it has been found that, despite the considerable homogeneity in their gross structural organization (**Figure 1**), substantial differences exist in their biophysical properties and gating behavior, sometimes even between members of the same channel subfamily. Thus, when submitted to constant depolarizing pulses to positive voltages from negative resting potentials, both eag1 and eag2 channels mediate outward-rectifying K^+^ currents that do not apparently inactivate. Furthermore, they show a prominent delay and slowing down of the activation when depolarization steps are preceded by negative prepulses, an effect (often misidentified as Cole-Moore effect, but see Hoshi and Armstrong, 2015) strongly dependent on extracellular Mg^2+^ and that sets the basis for their action under high frequency stimulation, since the activation is extremely slow in response to the initial stimuli, but speeds up with subsequent ones and, eventually, reaches a fairly fast activation which results in the current “appearing” only after a number of stimuli (Mortensen et al., 2015). Furthermore, both eag1 and eag2 are inhibited by binding the Ca^2+^-calmodulin (Ca^2+^-CaM) complex to several intracellular channel sites at the amino and carboxy termini. Nevertheless, clear differences in voltage dependence of activation are also exhibited by eag1 and eag2, since a much more positively shifted V_1/2_ value and a steeper slope of the activation curve are observed in eag1 as compared with eag2 channels (reviewed in Bauer and Schwarz, 2018).

Unlike the eag subtype channels, all erg channels inactivate and show unusual gating properties that allow them to act as crucial regulators of cell excitability (Bauer and Schwarz, 2018). Thus, erg channels show a slow activation overlapping with a rapid and voltage-dependent inactivation, leading to limited levels of outward current upon depolarization. Indeed, the steady-state erg current amplitude gets increasingly smaller at larger depolarizations due to more pronounced inactivation. At negative repolarization voltages, erg channels reopen due to their rapid recovery from inactivation before closing at a slow rate, leading to the appearance of prominent tail currents (Trudeau et al., 1995; Sanguinetti et al., 1995; Smith et al., 1996; Schönherr and Heinemann, 1996; Spector et al., 1996; Wang et al., 1997; Viloria et al., 2000). This peculiar kinetic combination, opposite to those of other voltage-dependent K^+^ channels, makes erg channels operate as inward rectifiers, although they have the six membrane-spanning domains and the VSD plus PD architecture typical of the depolarization-activated channels. Nevertheless, differences in gating behavior are also observed between the different erg channels. Thus, erg2 channels activate even more slowly than erg1, and erg3 channels show faster activation and deactivation but less inactivation, making them the weakest inward rectifiers of the group. Additionally, the erg2 activation curve is shifted to the right and that of erg3 to the left as compared to that of erg1 (Bauer and Schwarz, 2018). The physiological relevance of a population of inactivating voltage-dependent channels is determined by the so-called “window-current”, a bell-shaped curve resulting from the overlap of activation and inactivation curves that represents the steady-state fractional open probability as function of membrane potential, and defines the range of potentials at which the channels remain conductive (Becchetti et al., 2002; Bauer and Schwarz, 2018). Therefore, due to the aforementioned kinetic differences, a larger window-current covering a much broader voltage range is exhibited by erg3 channels as compared with erg1 (Bauer and Schwarz, 2018).

Differences in functional behavior are also found between members of the elk subtype channels. Thus, whereas no inactivation is present in elk1 and elk3, a clear inactivation is observed in elk2, that appears to be shifted to much more depolarized values than those of the erg subtype channels. Also, both elk1 and elk3 activate at more negative voltages, deactivate much more slowly and lack the typical Cole-Moore-like effect encountered in the non-inactivating channels of the eag subtype (Bauer and Schwarz, 2018). In our knowledge, the specific structural determinants for these differences in functional behavior remain unknown.

In summary, in spite of the obvious similarities in primary sequence (**Figure 1**) and in putative structural organization predicted for all EAG channels, the molecular basis of their kinetic characteristics and of the differences between them are not completely understood. It seems probable that whereas a common general architecture may determine the gating scheme used by the EAG channels and the physicochemical model involved in coupling voltage sensing to opening and closing the pore, subtle changes in such common structure and/or allosteric influences of protein domains, relatively distant from the central gating machinery, may crucially influence the gating process. The latest advances about this issue provided by the cryo-EM elucidation of eag1 and erg1 three-dimensional structures, and by both classical and more recent functional studies with different members of the EAG subfamily, are presented below.

## Mechanism(s) of VSD-PD Coupling for Gating the Non-Domain-Swapped Kv Channels of the EAG Family

As mentioned, the observation that Kv10.1 and Kv11.1 channels split at the S4–S5 linker maintain an almost unaltered voltage-dependent gating (Lorinczi et al., 2015), and the more recent demonstration of their non-domain-swapped architecture (Whicher and MacKinnon, 2016; Wang and MacKinnon, 2017), indicate that they must use a molecular mechanism for voltage-dependent activation different from the canonical S4–S5 linker lever-type of electromechanical VSD-PD coupling proposed for other voltage-dependent K^+^ (e.g., *Shaker*-like Kv1-9) and Na^+^ channels (Fernández-Mariño et al., 2018). It is important to note that, despite some subtle differences evidenced by the recently elucidated three-dimensional maps (Whicher and MacKinnon, 2016; Wang and MacKinnon, 2017), in the EAG channels the VSD structure, the location of the charge-carrying residues, the intra-VSD charge-charge interactions, and the extent of S4 translocation across the membrane, are similar to those of the classical *Shaker*-type Kv channels (Cheng and Claydon, 2012; Wang et al., 2013; Goodchild and Fedida, 2014). Also, whereas eag subtype channels typically activate (and deactivate) rapidly, some EAG channels (e.g., erg1/Kv11.1) exhibit a particularly slow activation (and deactivation) process (see above). In this sense, eag1 gating currents triggered by membrane depolarization and repolarization are rapid in onset and decay, but those from erg1 contain a major component an order of magnitude slower than the charge movements observed in eag1 (Piper et al., 2003; Bannister et al., 2005; Wang et al., 2013) and other Kv channels (Hesketh and Fedida, 1999; Bezanilla, 2000). Such a slow component could act, at least in part, as a rate-limiting step to explain erg1 slowness in pore opening and ionic current flow (Piper et al., 2003). However, even these slow reorganizations of erg1 VSD are not rate-limiting for pore gating, particularly over physiological voltages and timescales, since their voltage dependences and time constants for gating charge movement are still orders of magnitude separated from those of the activation of ionic currents (Wang et al., 2013; Goodchild and Fedida, 2014; Goodchild et al., 2015). Indeed, this has been interpreted as an indication that further transitions downstream of VSD rearrangements (e.g., some VSD-PD coupling steps and/or some allosteric interactions with distant structural elements that increase the time to pore opening, see below) are critically involved in the major erg1 pore opening delays (Goodchild and Fedida, 2014; Goodchild et al., 2015). Noticeably, some non-domain swapped ion channels are ligand-operated channels (e.g., SK, Slo2.2 and CNG channels, see above). It is obvious that in these cases binding of the regulator ligand to some specific domain(s) must allosterically control gating. However, participation of allosteric mechanisms on gating is not restricted to ligand-only operated channels, since they are also present in dual ligand-voltage regulated channels such as BK/Slo1 and HCN channels (see above). Thus, it is likely that these mechanisms may also influence gating in other non-domain-swapped, but genuinely Kv channels, such as eag1 (Kv10.1) and erg1 (Kv11.1). Indeed, binding of an intrinsic ligand to the eag1 CNBHD, is able to modulate activation in a way analog to cyclic nucleotide binding to other CNG family members (Zhao et al., 2017). Therefore, structural and functional evidences that the indicated allosteric interactions exist and their possible role in gating of non-domain-swapped eag1 and erg1 channels are considered next.

Early work with the EAG channels eag1, KAT1 and erg1, suggested that some of their amino terminal domains play an important role in setting the activation and deactivation gating characteristics, and pointed to a key role of interactions between them and the channels transmembrane core in this process, mainly with the carboxy end of the S4 segment or the S4–S5 linker. Thus, although structural alterations of the eag1 N-terminus impacted activation kinetics and abolished the Cole-Moore effect, these effects may be compensated by a single mutation of His343 (Terlau et al., 1997), which was initially suggested to be included in the S4 segment but more recently has been unequivocally located in the eag1 S4–S5 linker (Whicher and MacKinnon, 2016). In the same way, functional analysis of KAT-1 N-terminal deletion mutants, combined with S4 mutations, indicated that the N-terminus could also determine activation/deactivation kinetics, voltage dependence, and voltage sensitivity, probably by interacting with the voltage-sensing S4 segment (Marten and Hoshi, 1998). Initial studies with erg1 also indicated that regulation of deactivation involves the N-terminus and the S4–S5 loop, as if an interaction between these regions were essential for bringing the functional domains of the N-terminus into proximity with their targets in the hydrophobic core (Wang et al., 1998). In this case, missense mutations or chemical modification of the S4–S5 linker, mimic the effects of amino terminal deletion or missense mutations in the amino terminus, markedly increasing the rate of channel deactivation, and suggesting that the N-terminus of erg1 might interact with the S4–S5 linker to affect these changes (Sanguinetti and Xu, 1999; Chen et al., 1999). On the other hand, microinjection of a peptide corresponding to the entire eag domain into *Xenopus* oocytes expressing erg1 channels lacking this domain, or application of a peptide corresponding to the first 16 amino acids of the channel to excised membrane macropatches from oocytes expressing the same deleted erg1 channels, partially restored the slow deactivation gating properties that had been significantly accelerated by eag domain removal (Morais-Cabral et al., 1998; Wang et al., 2000). Moreover, expression of a recombinant eag domain (residues 1-135) in oocytes (Gustina and Trudeau, 2009), or transfected mammalian cells (Fernández-Trillo et al., 2011), causes complete recovery of the normal (slow) deactivation properties of N-terminally truncated erg1, lacking either the whole amino terminus or the eag domain. In this case, biochemical, functional and fluorescence resonance energy transfer (FRET) data directly demonstrated that the recombinant fragment interacts with the N-terminally truncated transmembrane channel core (Gustina and Trudeau, 2009; Gustina and Trudeau, 2011; Fernández-Trillo et al., 2011; Gustina and Trudeau, 2012; Gianulis et al., 2013; Codding and Trudeau, 2019). In this sense, a “master-switch” was proposed that maintains the erg1 slow rate of deactivation, requiring the simultaneous presence of all five Asp residues in the VSD. Binding of the N-terminal eag/PAS domain to the cytoplasmic S4–S5 linker would serve as such “master-switch” (Liu et al., 2003). Initial FRET experiments also suggested a very close proximity of the erg1 amino terminus to the central channel core, in good agreement with the proposed interaction(s) of the initial eag domain with the transmembrane channel structure, possibly at the level of the S4–S5 linker (Miranda et al., 2008; De la Peña et al., 2011; Barros et al., 2018). Indeed, this molecular organization has lately been corroborated by the eag1 and erg1 three-dimensional structures (Whicher and MacKinnon, 2016; Wang and MacKinnon, 2017). Finally, functional data indicate that two different domains regulating erg1 gating seem to be present in the N-terminus: a distal eag domain mainly controlling current deactivation, and a proximal domain regulating activation (Viloria et al., 2000). Removal of the eag domain from channels already lacking the proximal domain, noticeably reverses the effect on activation caused by the deletion of the proximal domain. This indicates that the effects of proximal domain removal are at least partially dependent on the presence of the eag domain, suggesting that an interaction of this domain with the channel core may be involved in alterations of activation caused by elimination of the proximal domain (Viloria et al., 2000).

It is important to note that direct interactions between the amino terminus and the transmembrane channel are not the only determinants for setting the activation and deactivation gating characteristics of *KCNH* channels. Indeed, the sequence similarity at the initial region of the C-terminus of these channels (from the end of the S6 helix to the end of the CNBHD) is also extendable to the cyclic nucleotide-dependent CNG and HCN channels (reviewed in Gustina and Trudeau, 2012; Morais-Cabral and Robertson, 2015; James and Zagotta, 2018). FRET data also suggested that, as proposed for the CNG and HCN channels (Zagotta et al., 2003; Craven and Zagotta, 2004; Hua and Gordon, 2005; Johnson and Zagotta, 2005; Taraska and Zagotta, 2007), the C-linker/CNBHD region “hangs” centrally below the transmembrane erg1 core, with the eag/PAS domain around its top and side surfaces, probably directed towards the gating machinery of the channel (Miranda et al., 2008). The recent cryo-EM structural models of both eag1 and erg1 (Whicher and MacKinnon, 2016; Wang and MacKinnon, 2017) confirmed that this region forms a tetrameric ring below the channel pore, which is surrounded by the eag domains docked to the outer surfaces of the CNBHDs. Interestingly, the predictions from the X-ray crystal structures of the isolated eag1 PAS-CNBHD complex (Haitin et al., 2013), and those based on homology modelling and protein-protein docking of the eag domain and the C-linker/CNBHD (Muskett et al., 2011), placed the N-tail interacting with the surface of the PAS region and/or the intracellular facing surface of the CNBHD, quite far from the elements of the gating machinery (e.g., the VSD/S4–S5 linker/gate interface) located at the intracellular plasma membrane surface (Muskett et al., 2011; Gustina and Trudeau, 2012; Vandenberg et al., 2012). This hypothesis was at odds with the idea of a direct interaction of the EAG channels N-tail with the transmembrane core (e.g., with the S4–S5 linker, see above) for regulation of gating. However, in some EAG channels the S4–S5 linker has been repeatedly proposed to act as an integrator of signals coming from other cytoplasmic domains to influence channel gating (Alonso-Ron et al., 2008; Ng et al., 2012; Tan et al., 2012; Zhao et al., 2017). In addition, a purified S4–S5 linker peptide (containing also residues from both S4 and S5) combines in solution with an isolated erg1 eag domain and causes a shift in the position of several amino acids in the eag domain NMR structure, suggesting that the peptide interacted with some region of such domain (Li et al., 2010). This possibility is also supported by results showing that in eag1 gating changes induced by small deletions in the initial portion of the N-terminus are compensated by a H343R point mutation at the S4–S5 linker (Terlau et al., 1997; Whicher and MacKinnon, 2016, see above). Finally, the observation that a disulfide cross-linkage can be formed between pairs of cysteine residues introduced in the erg1 N-tail and the S4–S5 linker, provided a more direct demonstration of a close physical proximity between both regions (De la Peña et al., 2011). Further analysis by the cysteine mutagenesis and disulfide chemistry approach suggested that, at least in some conformational state(s) of erg1, the flexible N-tail gets close to both the S4–S5 linker and some surface(s) of the C-linker/CNBHD domain relatively distant in the primary structure (De la Peña et al., 2013; De la Peña et al., 2015). Noticeably, whereas the N-tail region was disordered in the crystal structures of the erg1 eag domain, the solution structure solved by NMR spectroscopy indicated that the N-tail is structurally independent from the PAS region and contains a flexible initial segment of around 12 amino acids that form a highly dynamic, extended structure, followed by a helical element up to residue 25 (Li et al., 2010; Ng et al., 2011; Muskett et al., 2011). These data also provided a basis for the proposal that the flexible N-tail dynamically changes its position and/or orientation long enough to be able to interact with the VSD and the S4–S5 linker or the C-linker/CNBHD (De la Peña et al., 2013; Ng et al., 2014; De la Peña et al., 2015). Indeed, albeit indirectly, additional support for this idea was provided by docking the ensemble of the twenty erg1 eag-domain structures obtained by NMR (Li et al., 2010; Ng et al., 2011; Muskett et al., 2011; PDB codes 2L4R, 2L0W and 2L1M), against the C-linker/cNBD crystal structure of HCN2 (Zagotta et al., 2003; Haitin et al., 2013; PDB code 1Q5O), also docked below an erg1 transmembrane core structure homologous to that of Kv1.2 (Long et al., 2005; Long et al., 2007; PDB code 2A79). This opens the possibility that the flexibility of the N-terminal tail could allow it to adopt several positions from the bottom surface of the cNBD up to the S4–S5 linker (De la Peña et al., 2013; Morais-Cabral and Robertson, 2015). It also led us to propose the unifying hypothesis that a dynamic network of interactions may exist in EAG channels, involving the N-terminal tail, the S4–S5 linker and the C-terminal portion of S6, as well as other more distant cytoplasmic regions such as the PAS region and the C-linker/CNBHD (De la Peña et al., 2011; De la Peña et al., 2018a; Barros et al., 2018). If such a network constitutes an essential part of the gating machinery itself, only a regulator of the gating process(es) remains to be established. As also suggested by other authors (Morais-Cabral and Robertson, 2015), it is possible that the complex between the PAS and the C-linker/CNBHD domains could serve as an anchor to properly place and orientate the distal N-tail, that may constitute an important regulator of channel gating kinetics. This would also imply that changes in the conformation of either of these domains, or modifications of the interactions between them (Gustina and Trudeau, 2009; Gustina and Trudeau, 2011; Gianulis et al., 2013; Perissinotti et al., 2018; Codding and Trudeau, 2019), could result in gating changes due to alterations in the C-linker assembly, the relative orientation of the PAS and CNBHD domains, or the position of the N-tail (Morais-Cabral and Robertson, 2015; Perissinotti et al., 2018).

## New Structural and Functional Data Regarding Allosteric Influences of Cytoplasmic Domains in VSD-PD Coupling and Regulation of EAG Channels Gating

As indicated above, and mainly derived from important improvements in single particle cryo-EM, the architectural organizations of multiple ion channels pertaining to the named “S4-pore-loop” or “six-transmembrane domain one-pore domain” (6TM1P) group (Yu et al., 2005; Gutman et al., 2005; Ashcroft, 2006; Lau et al., 2018; Barros et al., 2019) have been determined. These findings led to the realization that, as initially demonstrated for eag1 and later confirmed for erg1 (Whicher and MacKinnon, 2016; Wang and MacKinnon, 2017), the overall architecture of the EAG channels subfamily differs from that of other Kv channels. Thus, the transmembrane core of these channels exhibits a non-domain-swapped molecular organization, in which the VSD of every subunit of the tetramer contacts the PD of the same polypeptide (**Figure 3**). This seems to preclude the possibility that the very short S4–S5 linker that connects both protein modules could act as a mechanical lever, like in domain-swapped Kv channels, that transmits the voltage-triggered reorganizations in the VSD (e.g., the movements of the S4 helix), to pull the S6 helices of neighboring subunits and open the channel gate at the bottom of the PD (Lu et al., 2002; Long et al., 2005; Long et al., 2007; Blunck and Batulan, 2012; Chowdury et al., 2014). The finding of such structural features also helped to understand previous data demonstrating that co-expressing N-terminal and C-terminal halves of these proteins separated at the S4–S5 linker (S4–S5 split channels) generated an almost unperturbed voltage-dependent activation gating (Lorinczi et al., 2015), further suggesting that in this type of Kv channels an alternative mechanism of voltage-dependent gating should exist. On the other hand, this opened some questions concerning the alternative molecular mechanism(s) and/or specific variations involved in voltage-dependent gating of these entities, and requested the possible contribution of additional structural and functional data, for example using split channels as an experimental tool, to better understand such new molecular mechanism(s).

In the solved cryo-EM structures of eag1 and erg1 the VSDs are in the depolarized/activated conformation, since they were obtained at a nominal voltage of 0 mV. Indeed, the observed structures of both VSD domains are almost identical (Whicher and MacKinnon, 2016; Wang and MacKinnon, 2017). However, an important difference between them is that whereas the erg1 intracellular gate at the S6 helical bundle is open, the eag1 structure shows the pore closed due to the inhibitory effect of the Ca^2+^/CaM complex bound to the cytoplasmic face of the channel (Whicher and MacKinnon, 2016; Wang and MacKinnon, 2017). The overlap of both pore structures indicates that they start to significantly deviate at a glycine gating hinge of the S6 helix located below the selectivity filter, corresponding to the residues G648 in hERG and G460 in eag1 (Thouta et al., 2014; Wang and MacKinnon, 2017). A similar gating hinge has been observed in an equivalent position in other classical domain-swapped “*Shaker*-type” Kv channels (Jiang et al., 2002). On the other hand, extensive anti-parallel contacts between the S5 and S6 pore helices, and interactions of the intracellular end of S4 with S5 and/or the intracellular portion of S6 and the C-linker, are observed in the EAG channels structures. Altogether, these facts led to propose that in these channels, the inward and centric displacement of S4 would close the S6 helical gate, allowing the VSDs to push and compress the S5 helices and transmit force through the S5-S6 interface (Whicher and MacKinnon, 2016; Wang and MacKinnon, 2017). The possibility that the movement of S4 could allow it to interact with the C-linker to bend the S6 helix and close the channel, in a way similar to that imposed by binding of Ca^2+^/CaM, has been also proposed (Whicher and MacKinnon, 2016; Wang and MacKinnon, 2017). Interestingly, in all cases an intact S4–S5 linker would not be necessary for VSD-PD coupling (Lorinczi et al., 2015; De la Peña et al., 2018a).

Unfortunately, until the structures can be determined with the VSDs in both the up and down conformations, it would remain unclear if the Ca^2+^/CaM-induced closed conformation of the eag1 pore is also representative of the closed state of both channels in response to changes in the VSD conformation triggered by membrane depolarization. Thus, the need for the S6 glycine kink is contradictory with previous data showing that, since in erg1 the S6 helices are inherently flexible, the S6 glycine residues are only required for the tight packing of the channel helices, but not as gating hinges for voltage-dependent activation (Hardman et al., 2007). On the other hand, in the erg1 open structure (Wang and MacKinnon, 2017), the S4–S5 linker residue Tyr542 seems to be directed towards the channel core, far from the initial residues of the N-tail to which it can be disulfide cross-linked, preferentially in the closed conformation, when cysteine pairs are introduced in both places (De la Peña et al., 2011). Also, the spatial locations of some residues in the C-linker (e.g., Cys723) do not seem optimal to establish disulfide bridges with N-tail engineered cysteines, as previously observed (De la Peña et al., 2013; De la Peña et al., 2015). Although it is possible that some of these cross-link results may be biased due to the introduction of cysteines in the putatively interacting positions, bringing on some alterations in the positioning or relationship between them, it seems clear that further functional and structural data with the VSD in both depolarized and hyperpolarized conformations, would be necessary to ascertain if the mentioned Ca^2+^/CaM-induced eag1 closed state corresponds to that achieved through voltage sensor reorganizations triggered by changes in membrane voltage.

During peer review of this manuscript, two seminal works were published concerning the mechanism of hyperpolarization-dependent opening of the non-domain-swapped HCN channels. Using a combination of long molecular dynamics (MD) simulations with the depolarized human HCN1 cryo-EM structure (Lee and MacKinnon, 2017) as template, as well as functional studies with HCN-eag chimeras, a hyperpolarization-induced break in the S4 helix of the VSD was observed. This break originated two sub-helices and placing the lower sub-helix in an orientation almost parallel to the membrane plane as a surrogate S4–S5 helix (Kasimova et al., 2019). The breaking transition seemed to be important for HCN1 hyperpolarization-dependent activation. Strikingly, the hydrophobicity of the amino acid following the breakpoint determined the gating polarity of some chimeric channels, changing it from a depolarization- to a hyperpolarization-dependent activation, opening the possibility that divergence of both types of channels could have occurred through a single point mutation in the S4 segment (Kasimova et al., 2019). The presence of the interfacial S4 sub-helix following the aforementioned S4 helix break, has been demonstrated in the recent cryo-EM structure of the HCN1 channel with the VSD chemically trapped in a hyperpolarized conformation by reversible, metal-mediated cross bridging (Lee and MacKinnon, 2019). Leaving apart the differences between their gating polarity, although both the HCN and eag channels share the same non-domain-swapped architecture, it is unclear if the two-helices break model also applies to members of the eag family because: (i) a S4 helix much shorter than that of HCN1 is observed in the structures of the eag1 and erg1 channels (Whicher and MacKinnon, 2016; Wang and MacKinnon, 2017), and (ii) quite small voltage sensor conformational changes seem to be induced in eag channels by changing the polarization of the membrane, as compared to those elicited in other Kv channels (Wang and MacKinnon, 2017). Further work would be necessary to ascertain if the two-helix break property is a common feature to other non-domain-swapped channels outside those of the HCN group.

One interesting feature in both the eag1 and the erg1 cryo-EM structures is that, despite the lack of domain swapping in their transmembrane core, the cytoplasmic regions do show a domain-swapped architecture. Thus, consistent with previous functional and structural studies (Stevens et al., 2009; Gustina and Trudeau, 2011; Gianulis et al., 2013; Haitin et al., 2013; Ng et al., 2014), the CNBHD of one subunit, which folds below the C-linker of the same subunit to which it is backbone connected, interacts at the same time with the N-terminal eag domain of the neighboring subunit (Whicher and MacKinnon, 2016; Wang and MacKinnon, 2017). Furthermore, in the case of eag1, binding of Ca^2+^/CaM to the PAS and CNBHD domains swapped between subunits establishes bridges between their N- and C-termini, that had been proposed to act as a molecular clamp to pull the two domains together translating the CNBHD interacting with the CaM toward the neighboring PAS (Whicher and MacKinnon, 2016; Barros et al., 2019). According to this proposal, since the CNBHD is connected to S6 *via* the C-linker, its movement toward the PAS domain would cause a rotation of both the C-linker and S6 to induce a 55° bend in a direction that tightens the helical bundle that forms the intracellular gate, inducing pore closure independently of transmembrane voltage (Whicher and MacKinnon, 2016). Recent data with this channel also described some interactions between the VSD, CNBHD and eag domains, that serve to modulate voltage-dependent channel gating, participate in the Cole-Moore effect, and are essential for Ca^2+^/CaM inhibition (Whicher and MacKinnon, 2019). It is interesting to note that some structural and/or functional elements participating in these interactions have been regarded as important modulators of gating in other EAG channels such as erg1, even though it does neither exhibit a Cole-Moore effect nor Ca^2+^/CaM-dependent regulation. The question that now arises is: what specific and distinctive molecular mechanism(s) could be involved in the voltage-dependent gating process of these channels and/or its possible regulation by the cytoplasmic domains?

**Figure 4:**
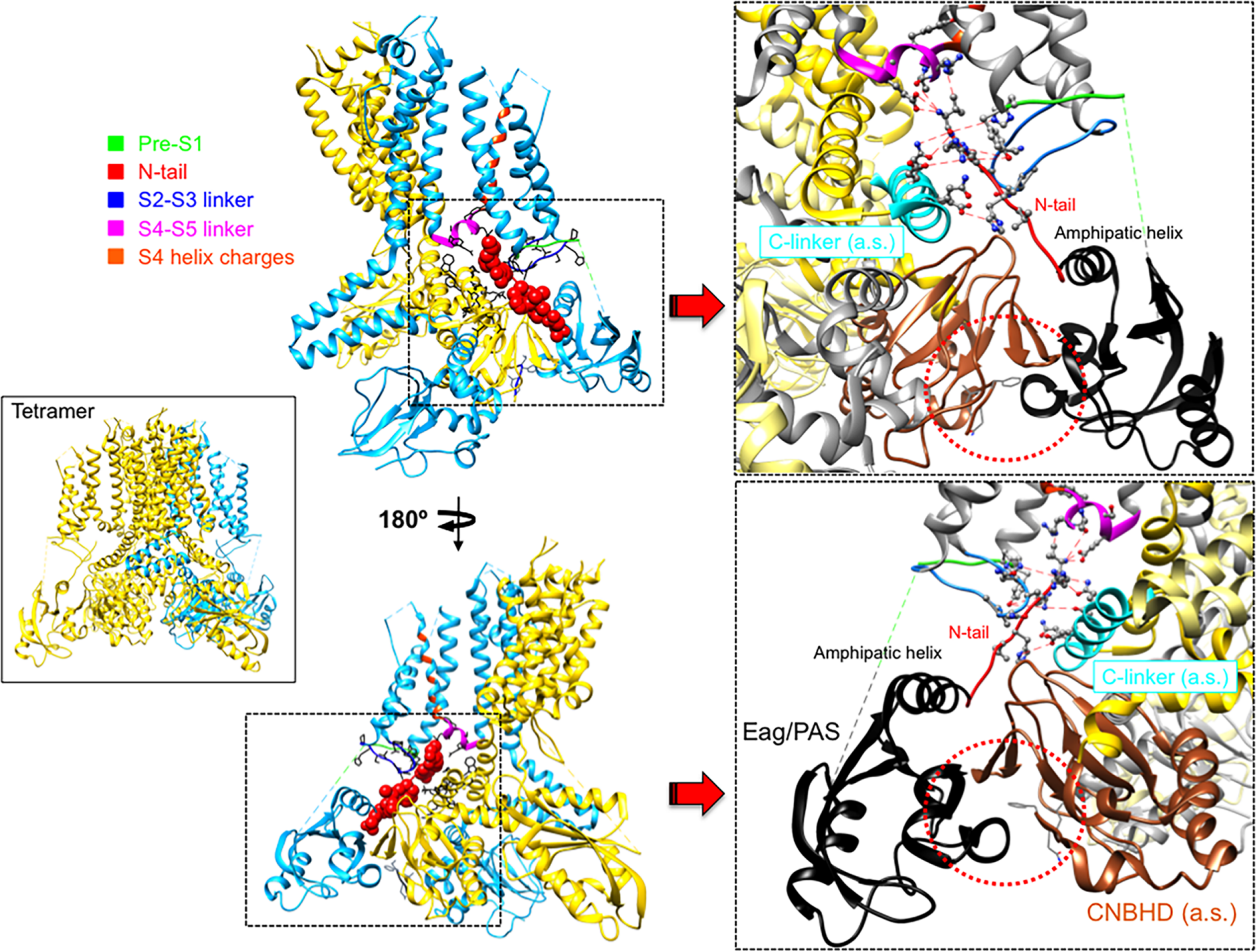
Close apposition of the erg1 (Kv11.1) N-tail with other cytoplasmic linkers of the channel and possible network of interactions that may dynamically contribute to modulate channel gating. *Left.* Lateral view of the erg1 tetrameric structure (Wang and MacKinnon, 2017; PDB code 5VA2) shown with ribbons. A highlighted subunit is colored in blue. *Center.* View of only two adjacent subunits in which some domains of the blue subunit are colored as indicated at the left high corner. Atoms corresponding to the amino terminal N-tail (red) are shown as spheres. Lateral chains of selected residues surrounding the space(s) around the N-tail position, pertaining to the pre-S1, S2–S3 and S4–S5 linkers of the same subunit, and the C-linker of the adjacent subunit, are presented as black sticks. Grey sticks at the bottom correspond to the residues that constitute the intrinsic ligand of the cyclic nucleotide-binding homology domain (CNBHD), occupying the ligand-binding pocket analogous to where cyclic nucleotides bind to the cyclic nucleotide binding domains in other channels. *Right.* Enlarged views of the regions inside the squares delimited by black dotted lines are shown. Lateral chains of selected residues protruding from structure ribbons are shown as ball and stick with oxygen and nitrogen atoms as red and blue small spheres, respectively. Ribbon sections are colored red (N-tail), green (pre-S1), blue (S2-S3 linker), magenta (S4–S5 linker) and cyan (C-linker of the adjacent subunit). Red dashed lines indicate interatomic distances ranging between 3.0 and 6.0 Å. Black and brown colors are used for ribbons corresponding to the *P*er-*A*rnt-*S*im (PAS) domain, and the CNBHD of the adjacent subunit (*a.s.*), respectively. Red dotted circles are used to mark the close proximity of these domains and the location of the CNBHD intrinsic ligand (gray sticks) toward the PAS domain surface. Structures were processed with UCSF Chimera (Pettersen et al., 2004).

An essential breakthrough provided by the new eag1 and erg1 structures was the direct confirmation that, through interactions between the PAS, C-linker/CNBHD and some other linkers at the VSD intracellular surface, the N-tail is directed towards the lower surface of the transmembrane core (Whicher and MacKinnon, 2016; Wang and MacKinnon, 2017). In the case of erg1, whose structure includes almost the entire N-tail, this region is positioned in close contact with both the S4–S5 linker of the same subunit and the C-linker of the adjacent subunit, respectively attached to the carboxy termini of the S4 and S6 helices (**Figure 4**). In addition, it also interacts with the intracellular S2-S3 linker and the S1 C-terminus of the same subunit (Whicher and MacKinnon, 2016; Wang and MacKinnon, 2017; Whicher and MacKinnon, 2019; Barros et al., 2019). The proximity of the N-tail to the S2–S3 linker and its possible implication in gating, would be also consistent with data showing that disruption of this linker causes alterations of erg1 gating, similar to those triggered by interrupting the S4 at its C-terminal end (De la Peña et al., 2018b). The S2-S3 linker of Drosophila eag has been proposed to play a role in the modulation of gating by Mg^2+^, perhaps through an electrostatic interaction with other intracellular domains or its interface with the transmembrane domains (Liu et al., 2010). On the other hand, the erg1 N-tail contact with residues H402 and T403 at the pre-S1 region could explain the gating perturbations triggered by mutations in these residues, similar to those caused by mutations in the N-tail itself (Phan et al., 2017). Furthermore, interaction and functional coupling of the erg1 S1 residue D411 with K538 at the inner end of S4 has been proposed (Zhang et al., 2005). In addition, it has been shown that interactions of D411 with lower S4 residues stabilize early closed states of the channel, and disruption of these interactions results both in: (i) faster rates of activation gating, and (ii) elimination of the fast component of gating charge movement and fluorescence changes associated to fast movement of S4 (Dou et al., 2017).

**Figure 5:**
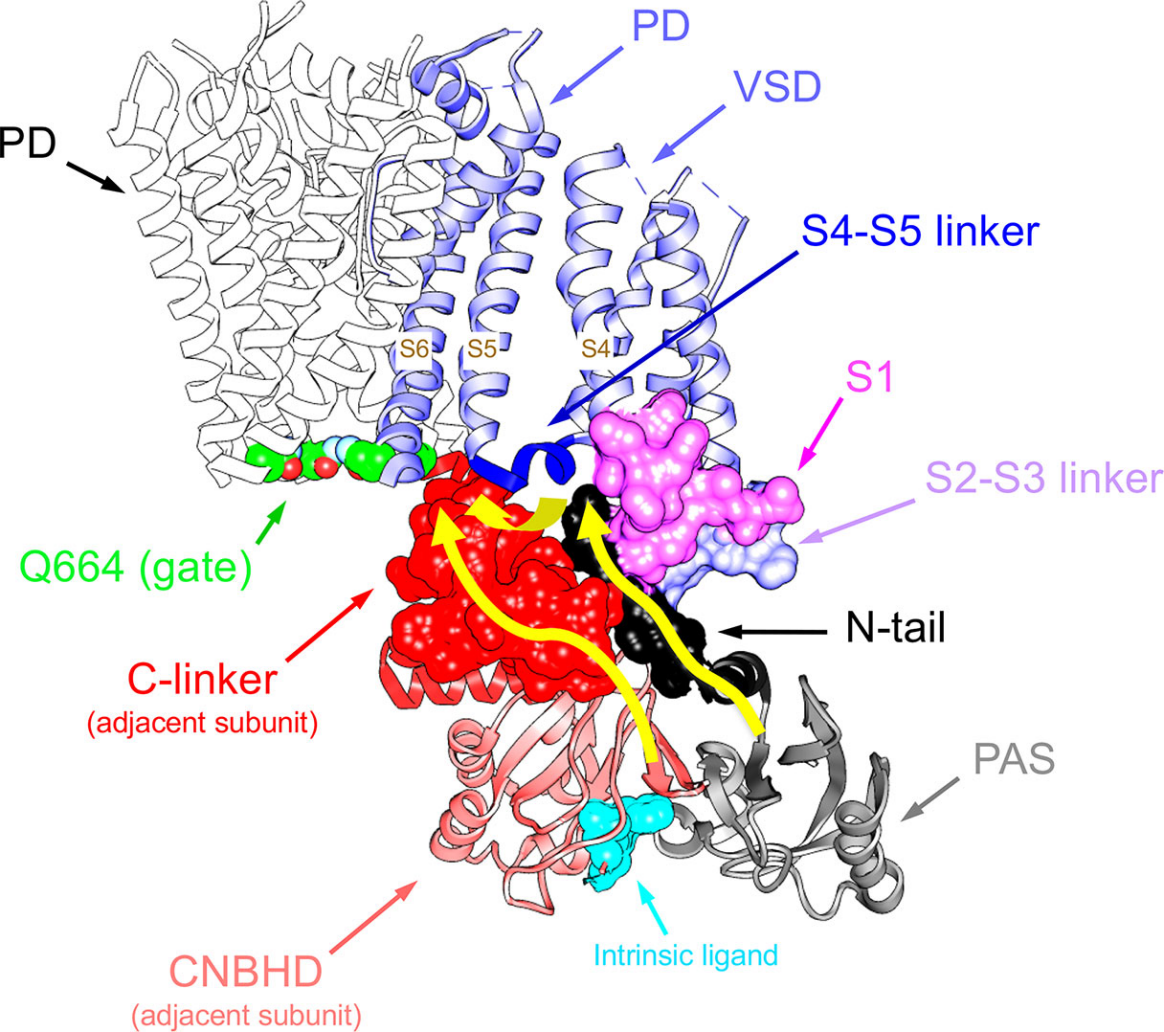
Pathways linking cytoplasmic domains and their possible structural reorganizations to VSD-PD coupling and functional operation of the erg1 (Kv11.1/hERG) channel gate. For clarity, a single voltage sensor domain (VSD) is shown attached to the tetramer of the pore domain (PD). The four Q664 residues that mark the place of the cytoplasmic gate at the S6 helix bundle are highlighted. Atoms corresponding to the amino terminal N-tail (black), the S2-S3 linker (blue) and the S1 C-terminus of the same subunit (magenta), and the C-linker of the adjacent subunit (red), are shown in space-filling mode. The positions of the PAS domain (grey) and the S4–S5 linker (dark blue), as well as the cyclic nucleotide-binding homology domain (CNBHD), domain of the adjacent subunit (pink), are also depicted. Atoms corresponding to the intrinsic ligand that binds to the cyclic nucleotide binding site of the CNBHD in the *P*er-*A*rnt-*S*im (PAS)–CNBHD interface between two adjacent subunits are presented as cyan spheres at the bottom. Yellow arrows highlight pathways for: (i) direct propagation of CNBHD conformational reorganizations to the pore gate *via* the C-linker, and (ii) allosteric coupling of the CNBHD to the PAS domain of the neighbouring subunit, and subsequent interplay of the PAS/eag domain with the lower portion of the VSD and the gate *via* the N-tail and the S4–S5 linker.

As indicated above, even before the realization that the S4–S5 linker of EAG channels is very short and structurally divergent from those encountered in other voltage-dependent channels, it was proposed that this linker acts as an integrator of signals coming from other cytoplasmic regions, such as the eag domain and the C-linker/CNBHD (Ng et al., 2012; De la Peña et al., 2018a). Thus, the direct linkage of the CNBHD with the C-linker of the same subunit, and through it to the pore gate at the cytoplasmic S6 helices bundle, provides a conduit for the effect of the CNBHD on the gate, as depicted in **Figure 5**. At the same time, the interaction of the CNBHD with the PAS region of the neighboring subunit, and that of the eag domain with the S4–S5 linker and the VSD *via* the N-tail (**Figure 5**), would provide a second way to allosterically control not only the gate itself, but also the VSD. Such dual pathway has been proposed to contribute to the role of the CNBHD and its intrinsic ligand in both the gating and the VSD movements of eag1 (Zhao et al., 2017). This fact would be consistent with results indicating that destabilization of the intrinsic ligand could lead to widespread changes in the gating assembly located below the VSD/S4–S5 linker/gate interface, *via* alterations in position, orientation, or flexibility of not only the C-linker/CNBHD complex, but also the interfacing N-tail/PAS domain from the neighboring subunit (Zhao et al., 2017). Noticeably, through a combination of mutagenesis, electrophysiology, and structural modeling using eag1 as a representative template for the erg1 closed pore, a similar interplay between N-tail, eag/PAS and VSD domains, S4–S5 and C-linkers, and CNBHD has recently been envisioned as an important contributor to erg1 gating kinetics (Perissinotti et al., 2018). These data further support the view that interactions between soluble domains and the transmembrane part of these channels are critical determinants of gating characteristics (Vandenberg et al., 2004; De la Peña et al., 2011; Fernández-Trillo et al., 2011; Vandenberg et al., 2012; Barros et al., 2012; Cheng and Claydon, 2012; Gustina and Trudeau, 2012; De la Peña et al., 2013; Morais-Cabral and Robertson, 2015; Perry et al., 2015; Vandenberg et al., 2017; De la Peña et al., 2018a; De la Peña et al., 2018b; James and Zagotta, 2018; Perissinotti et al., 2018; Barros et al., 2019). Interestingly, recent experiments with elk channels also indicate that the absence of either just the N-tail region, or the entire eag domain, causes the loss of the typical mode-shift or hysteresis (also called prepulse facilitation and voltage-dependent potentiation or VDP). This is a phenomenon shared by elk and erg channels, in which, due to a slow depolarizing voltage-dependent transition to a state favoring channel opening, a shift in the voltage dependence of activation to more hyperpolarized voltage is induced. This has been envisioned as an indication that rearrangement of an interaction between the eag domain and the CNBHD leads to a transition of the CNBHD intrinsic ligand from antagonist to agonist, thus acting as an allosteric modulator of channel gating during mode-shift (Dai and Zagotta, 2017; Dai et al., 2018).

In addition to the new structural data, the ability of EAG channels to non-covalently assemble from independent VSD and PD modules, maintaining their voltage-dependent gating, could provide clues about new molecular gating mechanisms, as well as a new tool to better understand the nature of the interactions and the dynamics involved in the gating process. Indeed, data comparing gating characteristics of eag1 and erg1 channels split at different points along the S4–S5 linker have yielded some valuable information about these issues. Thus, covalent breaks either at the C-terminal end of S4, or at the N-terminus of the S4–S5 linker, prevent closing of eag1 and yield constitutively active channels, while disconnecting the linker at its carboxy terminus from S5 does not disrupt closing, only leading to alterations of channel kinetics (Lorinczi et al., 2015; Tomczak et al., 2017). Interestingly, interrupting the channel at the C-terminus of S4 (or at the S4–S5 linker N-terminus) has little or no influence on the voltage dependence of VSD motion, even in those channels that show a constitutively active phenotype, although the modulation of the channel resting state by prepulse voltage and Mg^2+^ can be significantly affected. On the other hand, the constitutive activity of the channels split at the end of S4 is reverted to a wild type-like closure either introducing a variety of point mutations in the first residue (D342) of the split C-terminal demi-channel, or when a structural alteration is introduced at the amino terminus of the N-terminal demi-channel (Tomczak et al., 2017). While these results suggest that the S4–S5 linker may interact with the most N-terminal region of the channel, the interaction partner of D342 and the reason by which the amino terminal alteration causes a similar effect still remain to be established.

**Figure 6:**
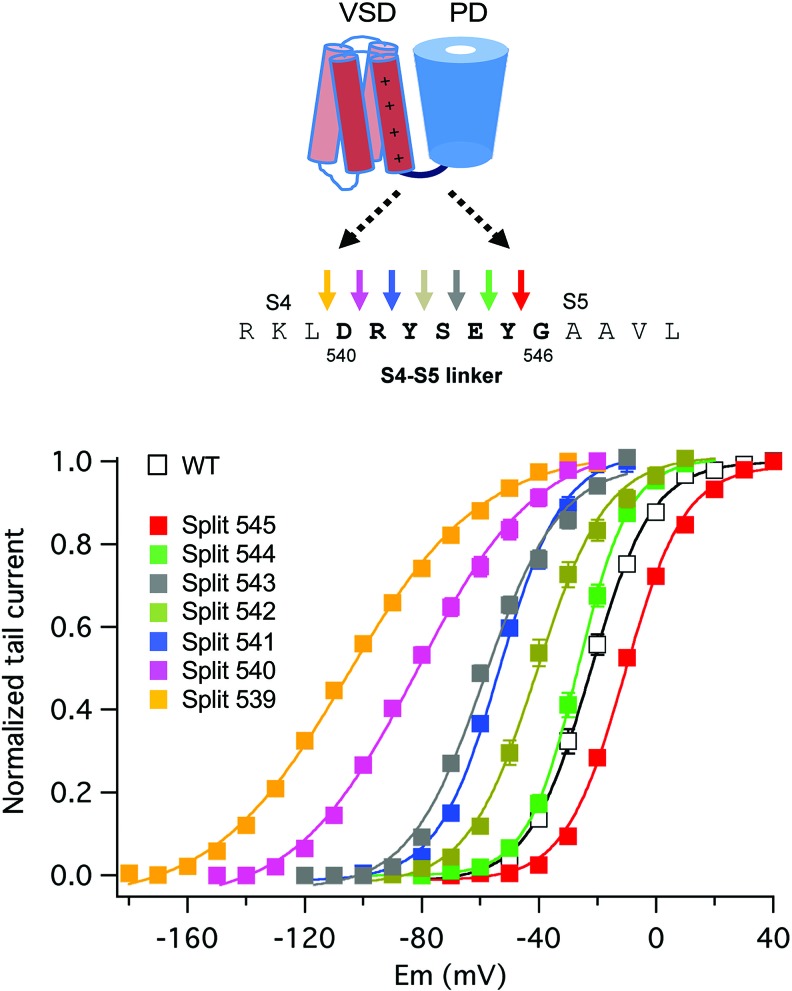
Gradual modification of activation voltage dependence caused by breaking the covalent linkage at different points along the S4–S5 linker sequence in split erg1 (Kv11.1/hERG) channels. A schematic view of the transmembranal core of a channel subunit at the top is combined with the sequence of the S4–S5 linker region and a set of coloured arrows for each covalent break used to generate the split channels listed below. Plots of normalized conductance/voltage relationships for the different splits are shown in the graph. Plots were obtained from tail current measurements upon repolarization. Tail currents were recorded in oocytes expressing the indicated channel variants, submitted to 1-s depolarization pulses at different potentials in 10 mV intervals from a holding potential of -80/-100 mV, followed by a repolarization step at -50 (splits 545, 544, 543 and 542), -70 (wild type/WT and split 541), -120 (split 540), and -140 (split 539) mV. Different repolarization potentials were used to ensure closing of the different constructs and proper quantification of tail current magnitudes. Continuous lines are Boltzmann fits to the data. For details see De la Peña et al., 2018a, from which the Figure has been reproduced under Creative Commons Attribution 4.0 license.

Similar experiments with erg1, progressively displacing the split position from the carboxy to the amino end of the S4–S5 linker, revealed a gradual modification of the activation gating characteristics of the split channels (**Figure 6**). Although no constitutively open channels were observed, channels split at the base of the S4 helix (around residue D540) exhibit a strong shift to hyperpolarized values in the voltage dependence of activation, a reduced ability to reach more distal closed state(s) and a reduced voltage dependence of both activation and deactivation gating (De la Peña et al., 2018a). This would also be consistent with previous data indicating i) the existence of an interaction between the bottom of S6 and residue 540 at the beginning of the S4–S5 linker (Tristani-Firouzi et al., 2002; Ferrer et al., 2006), ii) the generation a more unstable closed channel by mutating D540 to cysteine (Alonso-Ron et al., 2008), and iii) the stabilization of a closed conformation of the activation gate by covalently bridging D540 and the C-terminal S6 residue L666 (Ferrer et al., 2006). However, in contrast with the results obtained with eag1, the gradual negative shifts in erg1 activation voltage dependence observed when the split point was moved along the S4–S5 linker were paralleled by similar shifts in S4 voltage dependent motion across the membrane, suggesting that VSD and PD disconnection could also modify the voltage-dependent conformational reorganizations of the erg1 VSD (De la Peña et al., 2018a). Despite these functional differences, it seems that both in eag1 and erg1 an intact C-terminal end of the S4 helix (and/or the initial section of the S4–S5 linker) is important to reach a stable closed state, and that the VSD acts as an inhibitory module to close the channel at negative potentials (Tomczak et al., 2017; De la Peña et al., 2018a; James and Zagotta, 2018).

It should be noted that the need of S4 reorganizations to hold the permeation gate closed seems contradictory with the fact that, in both channels, expression of an isolated PD that appears to be effectively transported to the membrane (Tomczak et al., 2017), yields no detectable currents. On first sight, these results with only PDs expressed could be taken as an indication against the existence of a stable open state. However, it has been shown that besides the contribution of the VSD to the switch between resting and conductive channel states, this domain is also necessary for the PD to reach a conducting conformation (Tomczak et al., 2017). Indeed, conductive pore modules are not even detected upon expression of either isolated eag1 or erg1 PDs carrying mutations that disrupt the cytoplasmic gate (e.g. Q477P in eag1 or Q664P in erg1; Thouta et al., 2014; Tomczak et al., 2017) and that yield full-length channels permanently open at all voltages. This indicates that the energy landscape of the isolated PD can be different from that of the complete VSD/PD assembly, which may favor a different, preferentially opened basal conformation of the PD (Zhao and Blunck, 2016; De la Peña et al., 2018a). Albeit still speculative, the existence of a network of non-covalent interactions between the VSD, the PD, and the intracellular domains, playing a relevant role for this purpose, remains as an interesting possibility.

Finally, it is important to note that the proposed existence of a network of interactions involving the N-terminus, the S4–S5 linker and the final portion of S6, and other C-terminal regions, is also compatible with the named ligand/receptor (allosteric) model of voltage-dependent gating (Malak et al., 2017; Malak et al., 2019), recently shown to be shared by both erg1 and eag2 channels, in which the VSD and PD are weakly coupled *via* a ligand constituted by the S4–S5 linker and a part of S5, and a receptor, the C-terminal part of the S6 segment (Malak et al., 2017; Malak et al., 2019). The reason why the presence of a substantial part of the S5 helix is necessary for the soluble peptides used as ligands to stabilize the closed channel state (Malak et al., 2017) remains to be established. Strikingly, this mechanism was first proposed (Choveau et al., 2011) for KCNQ1 (Kv7.1), which shows domain-swapped transmembrane topology. Kv7.1 has a long α-helical S4–S5 linker (Sun and MacKinnon, 2017), and electromechanical VSD-PD coupling was therefore assumed to be similar to that encountered in other *Shaker*-like Kv channels, and not to that of the non-domain swapped EAG channels. It is important to emphasize also that, both in non-domain-swapped and in those Kv channels with prototypical domain-swapped organization in which a long S4–S5 linker can act as a lever for electromechanical VSD-PD coupling, the helices bundle that forms the pore gate is located at the bottom of S6 (Holmgren et al., 1998; Del Camino et al., 2000; Mitcheson et al., 2000; Del Camino and Yellen, 2001; Witchel, 2004; Webster et al., 2004; Del Camino et al., 2005; Swartz, 2005; Boulet et al., 2007; Wynia-Smith et al., 2008; Thouta et al., 2014). Therefore, even in the typical domain-swapped channels the covalent connection of the VSD and PD modules *via* S4–S5 linker would only directly track the S5 helix, making necessary some additional non-covalent interactions to gate them. In this sense, a non-canonical coupling pathway, based in non-covalent specific interactions between residues of the S4 and S5 helices, has recently been shown to participate in voltage-dependent activation gating of the prototypical Drosophila *Shaker* channel (Fernández-Mariño et al., 2018). Moreover, the possibility that the amino terminal end of the eag2 channel modulates the S4–S5 linker interaction with S6 and, as a consequence, that a hypothetical ligand/receptor gating mechanism exists, has been considered. Unfortunately, as previously shown with erg1 channels split at the S4–S5 linker, deletions of amino terminal sequences of eag2 obliterated by themselves the functional expression of the channels, complicating the possible interpretation of the data (Malak et al., 2019). In any case, it is tempting to speculate that the existence of allosteric components for modulation of gating, documented in Kv10-12 (EAG) channels, but also in other Kv relatives (Barros et al., 2019), constitutes a way to provide evolutionary functional diversification to a common and particularly successful 6TM1P molecular channel design.

In summary, the evidences compiled here point to the existence of a global and (perhaps more importantly) dynamic network of interactions involving the N-tail and the PAS and proximal domains at the channel amino terminus, the carboxy end of S4 and the S4–S5 linker, the C-terminal portion of helix S6, and the carboxy terminal C-linker and CNBHD domains, that allosterically contribute to modulate EAG channels gating (De la Peña et al., 2011; Fernández-Trillo et al., 2011; De la Peña et al., 2013; De la Peña et al., 2015; De la Peña et al., 2018a; De la Peña et al., 2018b; Barros et al., 2019; Whicher and MacKinnon, 2019). Nevertheless, the functional consequences of these interactions on activation/deactivation gating, and even on channel inactivation, can vary according to the channel type, the relative positioning of the N- and C-terminal regions, and the presence of additional auxiliary subunits. Noticeably, despite recent advances provided by atomic models of many channels, including some members of the EAG group, obtaining mechanistic insights from these structures remains somehow challenging. Therefore, to gain a better understanding of these complex but thrilling issues, further work would be still necessary in which the more static structural view is complemented with additional dynamic information provided by mutagenesis, combined functional and/or fluorometric assays such as voltage-clamp fluorometry, kinetic modelling and *in silico* molecular dynamics simulations.

## Author Contributions

All authors contributed to literature collection, interpretation and integration, and to writing the manuscript.

## Conflict of Interest

The authors declare that the research was conducted in the absence of any commercial or financial relationships that could be construed as a potential conflict of interest.
